# Examining the chronicity of suicidal thoughts and behaviors among Australian males

**DOI:** 10.1007/s00127-026-03172-7

**Published:** 2026-07-08

**Authors:** Tilahun Haregu, Shaosen Zhang, Jesse Young, Gregory Armstrong

**Affiliations:** 1 Centre for Mental Health and Community Wellbeing, Melbourne School of Population and Global Health, The University of Melbourne, Melbourne, Australia; 2 Institute for Mental Health Policy Research, Centre for Addiction and Mental Health, University of Toronto, Toronto, Ontario Canada; 3 Dalla Lana School of Public Health, University of Toronto, Toronto, Ontario Canada; 4 School of Population and Global Health, The University of Western Australia, Perth, Western Australia Australia; 5 Monash Addiction Research Centre, Eastern Health Clinical School, Monash University, Melbourne, Victoria Australia; 6 Centre for Epidemiology and Biostatistics, Melbourne School of Population and Global Health, The University of Melbourne, Parkville, Victoria Australia; 7 Justice Health Group, Curtin School of Population Health, Curtin University, Perth, Western Australia Australia; 8 Centre for Adolescent Health, Murdoch Children’s Research Institute, Parkville, Victoria Australia

**Keywords:** Chronicity, Suicidal ideation, Prevalence, Risk factors

## Abstract

**Purpose:**

Research on chronic suicidal thoughts and behaviors (STB) in Australia is limited. This study aimed to determine the prevalence and identify predictors of chronic STB among Australian males.

**Methods:**

A nine-year retrospective cohort study was conducted, analysing data from the first four waves of the Ten to Men Study. The study included 3,070 males who reported lifetime STB at Wave 1. Chronic STB was defined as the presence suicidal thoughts, plans, or attempts over subsequent waves. Modified Poisson and mixed-effects Poisson regression models were used to identify predictors.

**Results:**

The prevalence of chronic suicidal thoughts and attempts were 34.5% and 13.6%, respectively, with an overall chronic STB prevalence of 37.2%. At Wave 2, depression (IRR = 1.59; 95% CI [1.28, 1.98]; *P* < 0.001), homosexuality (IRR = 1.50; 95% CI [1.06, 2.14]; *P* = 0.023), and alcohol use disorder (IRR = 1.21; 95% CI [1.01, 1.46]; *P* = 0.044) were significantly associated with chronic STB. Over nine years, depression (IRR = 2.57; 95% CI [2.05, 3.22]; *P* < 0.001), alcohol use disorder (IRR = 1.53; 95% CI [1.27, 1.85]; *P* < 0.001), disability (IRR = 1.43; 95% CI [1.12, 1.82]; *P* = 0.004), and marijuana use (IRR = 1.33; 95% CI [1.05, 1.67]; *P* = 0.016) were key predictors.

**Conclusion:**

Among Australian men who report lifetime STB, more than one in three experience chronic STB over a nine-year period. Depression, alcohol use disorder, disability, illicit substance use, and socioeconomic factors emerged as primary predictors. Further research is needed to explore intervention strategies addressing these factors.

## Introduction

Suicide is a significant public health concern in Australia, being the leading cause of death among men aged 15–44 [1]. Approximately 75% of suicidal deaths in Australia occur among men [2]. Psychiatric disorders and harmful substance use (e.g., heavy alcohol and illicit drug consumption) confer a greater risk for suicidal behaviour worldwide. In Australia, higher suicide prevalence is found in more remote regions and Indigenous Australian communities [3, 4].

Suicidal behaviour is often conceptualized through the suicide cascade, a model that illustrates how various factors interact and escalate, ultimately leading from suicidal ideation to planning and, eventually, to an attempt or completion of suicide [5, 6]. The cascade represents a progression that typically occurs along a continuum. However, it is important to note that this progression is not always linear or inevitable. Several factors, such as an individual’s psychological state, external circumstances, and available support systems, influence this process [7].

A significant proportion of individuals who experience suicidal ideation, make suicidal plans, or attempt suicide will exhibit chronic suicidal thoughts and behaviors [8]. The 10-year follow-up of National Comorbidity Survey (NCDs) showed persistence of suicidal ideation among ideators at baseline to be 35%, while the prevalence for chronic suicidal planning and attempts were 21.2% and 15.4%, respectively [9]. Another study in a Danish Territory among those 15 years and older found that 30% of those who attempt suicide repeat the attempt within a year [10]. A Japanese study among suicide attempters admitted to emergency departments reported that the rate of chronic suicidal attempts in males within 5 years was 16.8% [11]. However, most of this evidence comes from clinical populations - individuals who are receiving treatment or care for mental health conditions, physical illnesses, or other medical conditions that may be associated with a higher risk of suicidal behaviour- and there is a lack of estimates on chronic suicidality and its predictors in longitudinal general population samples.

The causes of chronic suicidal thoughts and behaviors (STB) are multifaceted and complex, varying widely between regions, communities, and individuals. Some studies have identified factors associated with chronic suicidal behaviour, such as the lethality of suicide methods [12], the time elapsed since the previous suicide attempt [13], the number of previous suicide attempts [11], and the lack of mental health intervention after an attempt [10].

While studies generally consider relevant aspects contributing to chronic suicidal behaviour, they often focus on mental health disorders. The role of socioeconomic factors, such as income and employment, and substance use has not been explored. Moreover, factors known to be associated with a higher risk of any suicidal behaviour and especially relevant to Australia, such as rurality [14] and Index of Relative Socio-Economic Disadvantage and Aboriginal and Torres Strait Islander status [15, 16], are also absent from many studies on chronic suicidal thoughts and behaviors.

To enhance public health strategies aimed at reducing chronic STB in males, it is essential to investigate all potential risk factors, including those specific to Australia. Identifying and addressing these factors is crucial for optimizing suicide prevention measures among Australian males. Accordingly, our study aimed to (1) determine the rates of chronic STBs in Australian males; and (2) identify predictors of chronic STB using a nationally representative population-based cohort of males in Australia.

## Methods

### Study population

This study used data collected from the Ten to Men study, a large-scale cohort study focusing on the health of Australian males, which has been conducted in discrete survey waves since 2013. The Ten to Men cohort provides a nationally distributed snapshot of Australian males aged 10–55, capturing substantial diversity in geography, age, socioeconomic circumstances, cultural background, and health status. Its stratified, multi-stage sampling design ensures strong representation of urban and regional communities, a broad adolescent-to-midlife age range, and wide variation in education, employment, and wellbeing [17].

To date, four waves have been completed, with a unique questionnaire administered at each wave, involving the participation of over 16,000 men and boys in wave 1. Detailed information on the study’s design and methods have been published elsewhere [18, 19]. For the purposes of this study, we analyzed data from all four waves. Wave 1 was conducted from 2013 to 2014, while Wave 2 took place from 2015 to 2016. Waves 3 and 4 were conducted in 2020 and 2022/23, respectively. The questionnaire included sections covering various aspects of the participants’ lives, including socioeconomic factors, lifestyle choices, beliefs, and emotional well-being. Variables from various sections across these questionnaires were analyzed in relation to chronic STB. We conducted a retrospective cohort study involving males with history of lifetime suicidal thought, plan or attempt at Wave 1 and followed up in the subsequent three waves over a 9-year period.

### Study variables and measurement

#### Outcomes

In this study, suicidal thought and behaviour (STB) includes suicidal thought, plan and/or attempt as defined as a “Yes” response to questions regarding suicidal thoughts, plans, or attempts. At Wave 1, STB was assessed using lifetime questions about suicidal thoughts, plans, and attempts. In Waves 2–4, it was evaluated using questions about suicidal thoughts, plans, and attempts within the past 12 months. Chronic STB was then defined as the presence of STB (in the past 12 months) during follow-up in Waves 2–4 among males who reported lifetime STB at Wave 1. Chronic STB in this study includes both persistence and recurrence of STBs.

In this study, we examined chronic suicidality using suicidal thoughts and suicide attempts as the primary outcomes. These variables represent the core cognitive and behavioural dimensions of chronic suicidality and are the most stable and clinically consequential indicators over time. Suicide plans were not included because they represent an intermediate and often transient stage between thoughts and behaviour, are less consistently measured across waves, and do not align as directly with the chronicity construct central to our research aims.

### Predictor variables

Informed by interpersonal theory of suicide [6], we considered basic demographic factors, socioeconomic factors, health and substance use factors, and social and environmental factors as potential predictors of chronic suicidal thoughts and behaviors (STBs). Sociodemographic factors included age of the respondent at time of the surveys (in completed years), marital status (never married, divorced/separated/widowed, currently married), highest educational qualification (No completed qualification, certificate or diploma, bachelor degree, postgraduate, others), employment status (employed vs. not employed), and combined annual household income in Australian Dollars (categorized to low (< 40,000), middle (40,000–80,000), and high (above 80,0000)) based on the distribution of income within the sample to ensure adequate cell sizes across all four waves. These thresholds were selected for analytic stability rather than to represent socioeconomic advantage in the broader Australian context. Area-level factors included the remoteness of residence (categorized as major cities, inner regional and outer regional) and the Index of Relative Socio-Economic Disadvantage (IRSD), a measure used to assess the socio-economic disadvantage of a geographic area based on factors such as income, education, and employment, with lower scores indicating greater disadvantage [20]. Substance use factors considered were current smoking, alcohol use, and illicit substance use. Health-related factors included history diagnosis of depression and anxiety disorders in the past 12 months, and disability measured by Washington Group Short Set on social functioning [21]. Personal Wellbeing Index [22] and MOS (Medical Outcomes Study) social support score [23] were also included.

### Statistical analysis

We conducted all data analyses using Stata v18.0 [24]. Descriptive statistics were calculated for all measures. To ensure adequate statistical power across waves, we used STB, defined as the presence of suicidal thoughts, plans, or attempts in the past 12 months, as the outcome variable. To identify baseline predictors of chronic STB at Wave 2 among males who reported lifetime STB at Wave 1, we used a modified Poisson regression model. To assess predictors of chronic STB across Waves 2–4 over the nine-year follow-up period, we applied a mixed-effects Poisson regression model with an unstructured covariance matrix and participants included as a random effect. This model included only males who were followed across all four waves. The outcome was defined as the presence of STB in the 12 months preceding each of Waves 2–4, with predictor variables assessed during the same waves. Both the outcome and predictor variables were allowed to vary over time. Selection of variables for the final models was based on theoretical considerations, sample size, and an assessment of multicollinearity using the Variance Inflation Factor (VIF). To enhance statistical power, we combined suicidal thoughts, plans, and attempts into a single outcome variable (STBs) for the regression analysis. Results were presented as incidence rate ratios (IRRs) with corresponding 95% confidence intervals (CIs) and p-values.

## Results

### Participant characteristics

Our study included 3,070 males who reported any lifetime history of STBs at wave 1. The majority were married, employed, and resided in major cities. The prevalence rates for alcohol use disorder, a history of depression, and disability were 48%, 35%, and 14.9%, respectively. Additionally, around one-fifth of participants were diagnosed with anxiety disorders in the 12 months prior to wave 1. Detailed participant characteristics are presented in Table 1.

**Table 1:** Characteristics of the study participants by types of lifetime STBs at wave 1

Participants’ characteristics	All participants	Suicidal thought (*n* = 2,745)		Suicidal plan (*n* = 1,623)		Suicidal attempt (*n* = 802)		Any STBs (*n* = 3,070)	
	*n*(%)	*n*(%)	*P* value	*n*(%)	*P* value	*n*(%)	*P* value	*n*(%)	
10–17 yrs	2123(13.3)	198(7.2)	< 0.001	115(7.1)	< 0.001	46(5.7)	0.006	220(7.2)	< 0.001
18–29 yrs	3429(21.4)	691(25.2)		437(26.9)		209(26.1)		793(25.8)	
30–39 yrs	3685(23)	704(25.7)		391(24.1)		203(25.3)		770(25.1)	
40–57 yrs	6779(42.3)	1150(41.9)		679(41.9)		343(42.8)		1285(41.9)	
Marital status									
Never married	3644(26.5)	855(33.9)	< 0.001	535(35.8)	< 0.001	276(37.3)	< 0.001	968(34.3)	< 0.001
Divorced/separated/widowed	911(6.6)	290(11.5)		176(11.8)		115(15.6)		317(11.2)	
currently married	9189(66.9)	1375(54.6)		784(52.4)		348(47.1)		1534(54.4)	
Country of birth									
Australia-born	12,447(77.7)	2348(85.5)	< 0.001	1380(85)	< 0.001	649(80.9)	0.008	2592(84.4)	< 0.001
Overseas born	3574(22.3)	397(14.5)		243(15)		153(19.1)		478(15.6)	
Sexual orientation									
Heterosexual	13,317(93.1)	2400(89.7)	< 0.001	1414(89.1)	< 0.001	632(82.5)	< 0.001	2663(89.1)	< 0.001
Bisexual	209(1.5)	78(2.9)		57(3.6)		36(4.7)		85(2.8)	
Homosexual	229(1.6)	89(3.3)		59(3.7)		32(4.2)		103(3.4)	
Not sure and others	547(3.8)	108(4)		57(3.6)		66(8.6)		137(4.6)	
Highest educational qualification									
No completed qual	3416(26.3)	760(31.4)	< 0.001	480(33.5)	< 0.001	267(38.1)	< 0.001	848(31.5)	< 0.001
Certificate or diploma	5936(45.7)	1145(47.4)		651(45.4)		331(47.2)		1268(47)	
Bachelor degree	2115(16.3)	293(12.1)		168(11.7)		55(7.8)		335(12.4)	
Postgraduate	1247(9.6)	156(6.5)		86(6)		27(3.9)		175(6.5)	
Others	264(2)	64(2.6)		49(3.4)		21(3)		70(2.6)	
Employment status									
No	1963(14.5)	641(25.4)	< 0.001	404(27)	< 0.001	281(37.7)	< 0.001	709(25.1)	< 0.001
Yes	11,607(85.5)	1883(74.6)		1091(73)		464(62.3)		2115(74.9)	
Combined household income									
Less than 40 K	1333(11.3)	420(19.2)	< 0.001	255(19.9)	< 0.001	192(31.8)	< 0.001	459(18.9)	< 0.001
40k to 79.999 K	3205(27.1)	713(32.6)		424(33)		199(33)		788(32.4)	
80 K and above	7269(61.6)	1056(48.2)		604(47.1)		212(35.2)		1186(48.7)	
SEIFA quartiles									
First	4195(26.2)	884(32.2)	< 0.001	547(33.7)	< 0.001	323(40.3)	< 0.001	988(32.2)	< 0.001
Second	3983(24.9)	704(25.6)		392(24.2)		216(26.9)		780(25.4)	
Third	4029(25.1)	663(24.2)		413(25.4)		159(19.8)		750(24.4)	
Fourth	3814(23.8)	494(18)		271(16.7)		104(13)		552(18)	
Alcohol use disorder (Past 12 months)									
No	6947(60.3)	1111(51.3)	< 0.001	628(48.6)	< 0.001	268(47)	< 0.001	1245(51.7)	< 0.001
Yes	4574(39.7)	1053(48.7)		664(51.4)		302(53)		1165(48.3)	
Depression (past 12 months)									
No	12,687(87.2)	1666(62.6)	< 0.001	931(59)	< 0.001	409(53)	< 0.001	1931(65)	< 0.001
Yes	1861(12.8)	997(37.4)		647(41)		363(47)		1040(35)	
Current smoking									
No	10,886(79.7)	1668(66.1)	< 0.001	969(65)	< 0.001	407(54.6)	< 0.001	1887(66.9)	< 0.001
Yes	2769(20.3)	854(33.9)		522(35)		338(45.4)		934(33.1)	
Disability (WGS)									
No	13,679(93.1)	2289(84.7)	< 0.001	1333(83.3)	< 0.001	617(77.4)	< 0.001	2570(85.1)	< 0.001
Yes	1015(6.9)	415(15.3)		268(16.7)		180(22.6)		451(14.9)	
Marijuana use (past 12 months)									
No	10,821(80.7)	1697(68.5)	< 0.001	988(67.1)	< 0.001	479(65.4)	< 0.001	1919(69.2)	< 0.001
Yes	2583(19.3)	780(31.5)		485(32.9)		253(34.6)		856(30.8)	
Ecstasy/cocaine use (ever use)									
No	12,515(94.1)	2205(90.6)	< 0.001	1302(90.1)	< 0.001	638(89.1)	< 0.001	2477(90.8)	< 0.001
Yes	788(5.9)	228(9.4)		143(9.9)		78(10.9)		250(9.2)	
Anxiety disorders (past 12 months)									
No	12,431(91.8)	1909(77.6)	< 0.001	1098(75.2)	< 0.001	507(70.6)	< 0.001	2182(79.3)	< 0.001
Yes	1107(8.2)	552(22.4)		362(24.8)		211(29.4)		571(20.7)	
Remoteness of residence									
Major Cities	9371(58.5)	1508(54.9)	< 0.001	858(52.9)	< 0.001	423(52.7)	< 0.001	1697(55.3)	< 0.001
Inner Regional	3541(22.1)	688(25.1)		428(26.4)		227(28.3)		769(25)	
Outer Regional	3107(19.4)	549(20)		337(20.8)		152(19)		604(19.7)	
PWI score (Mean, SD)	70.3(17.2)	58.8(19.7)		57.9(20.2)		55.5(20.9)		59.6(19.5)	
MOS score (Mean, SD)	69.8(26.6)	61.7(28.6)		61.2(29.0)		59.2(28.7)		61.8(28.5)	

### Rates of chronic STBs

#### Any STBs

A total of 3,070 males (19.2%) reported any lifetime suicidal thoughts or behaviors (STBs) at wave (1) Among these, 579 (18.9%) reported STBs in the 12 months prior to wave (2) More than a third (37.2%) of males with a lifetime history of STBs at wave 1, who were followed up through wave 4, reported experiencing STBs during the 12 months preceding at least one of waves 2–4. The details of chronic STB rates are shown in Table 2 below.

**Table 2:** Rates of Chronic STBs among individuals exhibiting lifetime STBs at Wave 1 across subsequent waves

SN	Suicide variables	Wave 1 (lifetime STB, *n* = 14,779)	Wave 2 (STB in the past 12 months among those with the STB at wave 1 and followed up at wave 2)	(STB in the past 12 months at waves 2–4 among those with STB at baseline and followed up at all four waves)				
				None/unknown	Once	2 times	3 times	At least once
1	Suicidal thought	2745(17.1)	519(25.6)	687(65.5)	234(22.3)	85(8.1)	43(4.1)	362(34.5)
2	Suicidal plan	1623(10.1)	250(21.0)	420(68.4)	121(19.7)	45(7.3)	28(4.6)	194(31.6)
3	Suicidal attempt	802(5.0)	36(6.6)	184(86.4)	24(11.3)	*n* < 5(1.9)	*n* < 5(0.5)	29(13.6)
4	Suicidal behaviour	3070(19.2)	579(26.5)	706(60.7)	265(22.8)	111(9.5)	57(4.9)	433(37.2)

#### Suicidal thoughts

At wave 1, a total of 2,745 participants (17.1%) reported lifetime suicidal thoughts. Among these, 519 (18.9%) reported experiencing suicidal thoughts during the 12 months prior to wave 2. Overall, 362 (34.5%) of participants with lifetime suicidal thoughts at wave 1 who were followed up through wave 4 reported experiencing suicidal thoughts during the 12 months preceding at least one of waves 2–4.

#### Suicidal attempt

A total of 801 participants (5.0%) reported a lifetime suicide attempt at wave (1) Of these, 36 (4.5%) reported a suicide attempt in the 12 months prior to wave (2) Nearly 14% of males with a lifetime suicide attempt at wave 1, who were followed up through wave 4, reported experiencing a suicide attempt during the 12 months preceding at least one of waves 2–4.

### Predictors of chronic STBs

Among males with lifetime STB at Wave 1 who were followed up at Wave 2, a history of depression was strongly associated with chronic suicidal thoughts and behaviors (STBs), with those reporting depression having nearly 1.6 times higher rates of chronic STB at Wave 2. Homosexual males had a 1.5-fold higher rate of chronic STB. Males with alcohol use disorder and those with disabilities both had a 21% higher rate of chronic STBs compared to their counterparts. Homosexual men had 50% higher rates of chronic STB at Wave 2 as compared to heterosexual men (IRR = 1.50; 95% CI:1.06,2.14; *P* = 0.023). Men with “other” sexuality had significantly higher rates of chronic suicidality (IRR = 2.54; 95% CI:1.34, 4.80; *P* = 0.004). Other predictors included the SEIFA index, where individuals with lower socioeconomic disadvantage (third quartile) had higher rates of chronic STBs, as well as the Personal Well-being Index and social support, both of which were negatively associated with chronic STBs. Details of the model outputs are shown in Table 3 below.

**Table 3:** Predictors chronic STB at wave 2 among those with lifetime STB at wave 1

Baseline predictors	IRR	95% CI		*P* Value
Age categories (Ref: 18–29 years)				
30–39 years	0.91	0.69	1.18	0.468
40–57 years	0.95	0.73	1.24	0.721
Marital status (Ref: Never married				
Divorced/separated/widowed	0.77	0.52	1.13	0.182
Currently married	1.39	1.08	1.79	0.010
Country of birth (Ref: Australia-born)				
Overseas born	0.92	0.70	1.20	0.528
Sexuality (Ref: Heterosexual)				
Bisexual	1.08	0.55	2.10	0.825
Homosexual	1.50	1.06	2.14	0.023
Not sure	0.72	0.28	1.85	0.495
Other	2.54	1.34	4.80	0.004
Educational qual (Ref: High school or less)				
Certificate or diploma	0.87	0.70	1.08	0.199
Bachelor’s degree	0.72	0.53	0.99	0.043
Postgraduate	0.84	0.57	1.22	0.359
Others	0.71	0.37	1.35	0.293
Employment (Ref: No)	0.83	0.65	1.07	0.148
Combined household income (Ref: <40 K)				
40k to 79.999 K	0.88	0.66	1.18	0.409
80 K and above	1.11	0.83	1.48	0.492
SEIFA quartiles (Ref: First)				
Second	0.96	0.74	1.25	0.788
Third	1.29	1.01	1.63	0.037
Fourth	1.09	0.81	1.45	0.582
Alcohol use disorder (Ref: No)	1.21	1.01	1.46	0.044
Depression (past 12 months) (Ref: No)	1.59	1.28	1.98	< 0.001
Current smoking (Ref: No)	1.10	0.89	1.35	0.387
Disability (WGS) (Ref: No)	1.21	0.95	1.55	0.123
Marijuana use (past 12 months) (Ref: No)	0.83	0.66	1.04	0.099
Ecstasy/cocaine use (Ref: No)	1.31	0.94	1.82	0.106
Anxiety disorders (past 12 months) (Ref: No)	0.83	0.66	1.04	0.110
Remoteness of residence (Ref: Major cities)				
Inner Regional	1.11	0.90	1.39	0.332
Outer Regional	1.05	0.82	1.34	0.705
PWI	0.99	0.98	0.99	< 0.001
MOS	1.00	0.99	1.00	0.004

Outputs are from a modified Poisson regression model

Participants are males with lifetime STB at wave 1 and followed up at wave 2

Among males who reported lifetime STB at Wave 1 and were followed up across four waves over nine years, a history of depression was associated with a nearly 2.6-fold increase in the likelihood of chronic STB. Alcohol use disorder was linked to a 1.53-fold higher risk, while disability was associated with a 1.43-fold increase. Illicit substance use was associated with a 1.33-fold higher likelihood of chronic STB. Other longitudinal predictors of chronic STB included age, marital status, personal well-being index, and social support. Details of the model outputs are shown in Table 4 below.

**Table 4:** Predictors chronic STB overtime among those with lifetime STB at wave 1 and followed up through wave 4

Predictors (time varying)	IRR	95% CI		*P* value
Age in years	1.03	1.01	1.04	< 0.001
Marital status (Ref: Never married)				
Divorced/separated/widowed	0.87	0.66	1.16	0.346
Currently married	0.68	0.53	0.87	0.002
Country of birth (Ref: Australia-born)				
Overseas born	0.83	0.62	1.12	0.230
Educational qual (Ref: High school or less)				
Certificate or diploma	0.98	0.78	1.22	0.830
Bachelor’s degree	0.86	0.63	1.18	0.358
Postgraduate	0.83	0.60	1.16	0.282
others	1.22	0.72	2.09	0.460
Employment (Ref: No)	1.24	0.95	1.60	0.112
Combined household income (Ref: <40 K)				
40k to 79.999 K	1.05	0.79	1.40	0.727
80 K and above	1.06	0.80	1.40	0.697
SEIFA quartiles (Ref: First)				
Second	1.14	0.86	1.51	0.349
Third	1.10	0.83	1.47	0.504
Fourth	0.94	0.68	1.30	0.718
Alcohol use disorder (Ref: No)	1.53	1.27	1.85	< 0.001
Depression (past 12 months) (Ref: No)	2.57	2.05	3.22	< 0.001
Current smoking (Ref: No)	0.79	0.62	1.02	0.069
Disability (WGS) (Ref: No)	1.43	1.12	1.82	0.004
Marijuana use (past 12 months) (Ref: No)	1.33	1.05	1.67	0.016
Ecstasy/cocaine use (Ref: No)	1.10	0.74	1.64	0.622
Anxiety disorders (past 12 months) (Ref: No)	0.83	0.65	1.06	0.142
Remoteness of residence (Ref: Major cities)				
Inner Regional	1.09	0.86	1.38	0.471
Outer Regional	1.05	0.81	1.36	0.697
PWI	0.98	0.98	0.98	< 0.001
MOS	0.99	0.98	0.99	< 0.001

Outputs are from mixed effects Poisson regression model

Participants are males with lifetime STB at wave 1 and followed up until wave 4

## Discussion

We found that, among Australian men who report lifetime STB, more than one-third experience chronic STB over a nine-year period. The main predictors of chronic STBs were history of depressive disorders, alcohol use disorder, and disability. Additionally, social disadvantage, personal well-being, and social support played a role in the chronicity of suicidal behaviour.

Our findings are consistent with the findings of existing research which demonstrate a similarly high level of chronicity of such behaviour in more than a third of people with a baseline history of suicide ideation continuing to have it at some time over the next decade [9, 11]. A cross-national study demonstrated that 60% of transitions from ideation to plan and attempt occur within the first year after ideation onset [25]. This highlights the significant risk of eventual suicidal attempts in individuals with active suicidal thoughts, underscoring the need to not only study factors leading to suicidal attempts, but also those contributing to the chronicity of suicidal thoughts and planning. The same study found that almost all mental health disorders considered in the study, including depression and post-traumatic stress disorder, were found to be predictors of chronic suicidal behaviour [16].

Prior research has found that alcohol and drug use, depression, and disability are associated with STBs. Our study extends this by providing novel evidence that these risk factors are also linked to the chronicity of STBs. Depression, substance use, and alcohol use disorder had the largest effect effects on chronic STBs in our study. Depression is closely linked to chronic STB, with individuals experiencing recurrent or untreated depression at higher risk for persistent suicidal thoughts and actions. This interplay increases the likelihood of prolonged STB [8, 14, 26–28]. In our study, marijuana use significantly predicted chronic STBs. Repeated use of marijuana use often leads to depression, anxiety, and irritability during withdrawal, increasing the risk of chronic suicidality [29].

Homosexual men showed substantially higher rates of chronic suicidal thoughts and behaviours (STBs) at Wave 2, with rates approximately 50% higher than heterosexual men. Men identifying with “other” sexual orientation demonstrated even greater elevation, with rates more than twice those of heterosexual men. These findings align with extensive evidence that sexual minority groups experience elevated risk for suicidal distress, often attributed to minority stress processes, discrimination, and reduced access to affirming support services [30–32]. The particularly high rates among men in the “other” category may reflect additional vulnerabilities associated with less clearly defined or more socially marginalised identities.

Alcohol use disorder (AUD) is also strongly associated with chronic STB. AUD increases the risk of persistent suicidal thoughts and actions by exacerbating mental health issues like depression and anxiety, impairing judgment, and heightening impulsivity [9, 33, 34]. Similarly, disability is associated with higher rates of chronic STBs. This could be due to the psychological, social, and physical challenges it entails.

Our study has identified protective factors against chronic suicidal thoughts and behaviors (STBs). We found that males experiencing lower socioeconomic disadvantage, as indicated by being in the third SEIFA quartile, had higher rates of chronic STBs. This contrasts with previous research, which has typically reported a negative association between SEIFA and suicidal behavior [16, 35]. Additionally, personal well-being has been linked to a reduced risk of suicidal behavior [36, 37],, while social support has also been shown to have a protective effect [38, 39]. Our study builds on this evidence by demonstrating that these protective factors extend to the chronicity of suicidal behavior among males.

We observed a negative association between smoking and chronic suicidal thoughts and behaviours (STBs), even though this relationship did not meet the conventional *P* < 0.05 threshold. It has been well established that smoking increases the risk of suicidal ideation and attempts [40–42], and our prior analyses with the same data also reported a positive association between smoking and suicidal behaviour [41]. Additionally, research suggests that population-level smoking interventions may help reduce suicide risk [43]. The negative association observed in our study has resulted from adjusting for depression and substance use. Controlling for the relationship between mental illness, substance use and smoking, our analysis showed the independent effect of smoking on chronic suicidality. Nonetheless, this finding should be treated with strong caution, given the strength of prior findings on the positive association between smoking and suicide [40–42].

This study has some limitations. First, all data were self-reported, making recall bias and social desirability bias possible. Second, substantial dropout, which was associated with baseline STB, across the waves of the Ten to Men study may impact the generalizability of our findings. Due to this dropout, some subgroups, such as Aboriginal and Torres Strait Islander people, could not be included in the analysis due to insufficient statistical power. Stigma surrounding suicidal disclosure among men may have contributed to under-reporting of chronic STBs and to selective dropout across waves, particularly among those experiencing greater distress. As a result, prevalence estimates and longitudinal patterns may underestimate the true burden of chronic STBs in this population. Third, our definition of chronic STBs was broad, encompassing suicidal thoughts, plans, and attempts. However, it did not capture the transition from suicidal thoughts to plans and attempts over time.

## Conclusion

In conclusion, this study revealed that chronic STBs affect over a third of Australian males who have previously exhibited such behaviors. We identified four predictors of chronic STBs: depression, alcohol use disorder, disability, and illicit substance use. These findings emphasize the need for targeted interventions that address these factors to help reduce chronic STBs in this population. Further research is needed to explore how modifying these predictors may prevent future incidents of STB and contribute to more effective suicide prevention strategies.

## Data Availability

Data are available up on request from Australian Institute of Family Studies.
